# Therapeutic efficacy of a novel humanized antibody-drug conjugate recognizing plexin-semaphorin-integrin domain in the RON receptor for targeted cancer therapy

**DOI:** 10.1186/s40425-019-0732-8

**Published:** 2019-09-13

**Authors:** Xiang-Min Tong, Liang Feng, Sreedhar Reddy Suthe, Tian-Hao Weng, Chen-Yu Hu, Yi-Zhi Liu, Zhi-Gang Wu, Ming-Hai Wang, Hang-Ping Yao

**Affiliations:** 1 Department of Hematology, Zhejiang Provincial People’s Hospital, Hangzhou Medical College, Hangzhou, China; 2 Cancer Biology Research Center, Texas Tech University Health Sciences Center School of Pharmacy, Amarillo, TX USA; 3 Department of Pharmaceutical Sciences, Texas Tech University Health Sciences Center School of Pharmacy, Amarillo, TX USA; 4 State Key Laboratory for Diagnosis & Treatment of Infectious Diseases, First Affiliated Hospital, Zhejiang University School of Medicine, Hangzhou, China; 5 National Clinical Research Center for Infectious Diseases, First Affiliated Hospital, Zhejiang University School of Medicine, Hangzhou, China

**Keywords:** RON receptor tyrosine kinase, PSI domain, Monoclonal antibody, Humanization, Antibody-drug conjugates, Receptor internalization, Epithelial cancer, cancer stem cells, Tumor xenograft model, Therapeutic efficacy

## Abstract

**Background:**

Antibody-drug conjugates (ADCs) targeting the RON receptor, a tumorigenic factor contributing to cancer malignancy, has been considered as a novel strategy for cancer therapy. Here we describe a humanized antibody recognizing the RON plexin-semaphorin-integrin (PSI) domain with increased drug delivery capability for potential clinical application.

**Method:**

Monoclonal antibody PCM5B14 specific to the human and monkey RON PSI domain was generated and characterized by various immunological methods. Humanized antibody H5B14 was created by grafting PCM5B14 complementarity-determining regions into human IgG1/κ acceptor frameworks and conjugated with monomethyl auristatin E and duocarmycin to form two H5B14-based ADCs. Stability of H5B14-based ADCs in human plasma was measured using hydrophobic interaction chromatography. Various biochemical and biological assays were used to determine ADC- regulated RON internalization, cell viability, spheroid formation, and death of cancer stem-like cells. Efficacies of H5B14-based ADCs in vivo were validated using tumor xenograft models. Maximal tolerated doses of H5B14-based ADCs were established in mice.

**Results:**

H5B14 was highly specific to the human RON PSI domain and superior over other anti-RON ADCs in induction of RON internalization in various cancer cell lines tested. H5B14-based ADCS had a drug to antibody ratio of ~ 3.70:1 and were stable in human plasma with a minimal dissociation within a 10-day period. Functionally, H5B14-mediated drug delivery decreased cell viability at early stages with an average IC_50_ at ~ 20 nM in multiple cancer cell lines examined. H5B14-based ADCs also inhibited spheroid formation and caused death of cancer stem-like cells with RON^+^/CD44^+^/ESA^+^ phenotypes. In vivo*,* H5B14-based ADCs in a single injection inhibited tumor xenograft growth mediated by multiple cancer cell lines. Tumoristatic concentrations calculated from xenograft tumor models were in the range of 0.63 to 2.0 mg/kg bodyweight. Significantly, H5B14-based ADCs were capable of eradicating tumors at variable levels across multiple xenograft models regardless their malignant statuses. Toxicologically, H5B14-based ADCs were well tolerated in mice up to 60 mg/kg.

**Conclusion:**

H5B14-based ADCs targeting the RON PSI domain are superior in inducing RON internalization, leading to robust drug delivery and overall inhibition and eradication of tumors in multiple xenograft models. These findings warrant H5B14-based ADCs for clinical trials in the future.

## Introduction

Antibody-drug conjugate (ADC) targeting the RON receptor tyrosine kinase is a promising strategy for cancer therapy and currently is under intensive evaluation for potential clinical trials [1–6]. RON belongs to the MET proto-oncogene family, which contributes to cancer tumorigenesis, malignancy, and chemoresistance [7, 8]. Pathologically, RON is overexpressed in various types of cancers including colon, lung, breast, and pancreatic tumors [9–12]. Increased RON expression also is an indicator for the shortened survival of certain types of cancer patients [13]. At the cellular level, constitutive RON activation transduces signaling that promotes epithelial to mesenchymal transition leading to aggressive phenotypes [14–18]. Aberrant RON expression also is associated with production of truncated/splicing oncogenic variants [19–22], which facilitates cancer cell invasive growth and chemoresistance [19–22]. Clearly, the pathogenic features of RON provide the molecular basis for targeting RON for cancer therapy.

For the last decade, we have focused on development of RON-specific ADCs for cancer therapy [1–6]. By characterizing monoclonal antibodies (mAbs) specific to RON [23–25], we have selected lead candidates based on their antigen specificity, binding sensitivity, and suitability as biotherapeutics for ADC development [1–6]. Structurally, ADCs are composed of an antigen-specific antibody conjugated with a highly potent cytotoxin such as maytansinoid derivative 1 (DM1), monomethyl auristatin E (MMAE), and duocarmycin (DCM) through a chemical and/or protease cleavable linker [26, 27]. Various factors, including antigen expression, antibody-binding affinity, antibody-induced target internalization, cytotoxic compound selection, chemical linker property, all influence ADC pharmacokinetics and therapeutic efficacy [26, 27]. Thus, biochemical and pharmaceutical optimization of ADCs is essential for successful development of lead candidates for clinical trials and patient application.

Ligand-dependent activation causes RON endocytosis into cytoplasmic compartment [23, 24]. This effect also is observed upon antibody binding to the RON extracellular domains [1–6, 28, 29], which is the first step required for delivery of cytotoxic drugs for cancer cell killing. In this sense, selection of mAbs that are capable of inducting robust RON internalization for drug delivery is the key step for the validation of RON-targeted ADCs. The extracellular sequences in the RON β-chain contain a semaphorin (SEMA) domain followed by a plexin-semaphorin-integrin (PSI) domain and three immunoglobulin-like plexin and transcription (IPT) motifs [7, 8, 30]. The SEMA domain harbors a high-affinity ligand-binding pocket, which, upon ligand interaction, is capable of dimerizing RON for signaling transduction [31, 32]. The PSI domain serves as a wedge between the SEMA domain and IPT motifs and facilitates the formation of a RON homodimer with interface formed by the SEMA domain [31, 32]. In this sense, the PSI domain is responsible for the correct positioning of the ligand-binding site of RON. Studies also observe that mAbs binding to the PSI domain cause a rapid MET internalization by cancer cells [33]. This suggests that the use of mAbs to target the PSI domain to induce receptor internalization could be a critical pharmaceutical approach for drug delivery.

The study presented here is about humanized mAb H5B14 specific to the PSI domain in induction of RON internalization and to validate the efficacy of H5B14-based ADCs in inhibition and/or eradication of tumor xenografts in mouse models. We have previously shown that mAbs such as Zt/g4 specific to the SEMA domain is capable of induction of RON internalization [1–6]. Consistent with these observations, Zt/g4-based ADCs are effective in inhibition and eradication of xenograft tumors derived from colon, breast, lung, and pancreatic cancer cell lines [1–6]. Currently, the role of the PSI domain in regulating RON internalization is unknown. Availability of our mAbs specific to the RON PSI domain such as H5B14 provides an opportunity to test whether this mAb is suitable candidate for development of RON-targeted ADCs for clinical application.

## Materials and methods

### Cell lines, reagents, and animals

Pancreatic adenocarcinoma (PAC) ASPC-1, Panc-1, BxPC-3, colorectal cancer LoVo, HT-29, HCT116, SW620, breast cancer MCF-7, T-47D, MDA-MB-231, Du4475, and lung cancer H1993, H358, H2228 cell lines were from American Type Cell Culture (ATCC, Manassas, VA). Additional PAC cell lines FG and L3.6pl were provided by Drs. A.M. Lowy (University of California at San Diego, San Diego, CA) and G.E. Gallick (University of Texas M.D. Anderson Cancer Center, Houston, TX), respectively. All cell lines were authenticated in 2015 with cytogenesis analysis performed by ATCC. Stable NIH3T3 cells expressing human, monkey, or mouse RON were used as previously described [6, 19]. Mouse anti-RON mAbs Zt/g4, Zt/c1, Zt/c11, Zt/f2, Zt/f12, and rabbit IgG antibody R#5029 against the RON C-terminus were used as previously described [19, 23]. Female athymic nude mice at 6 weeks of age were from Taconic Biosciences [Granbury, NJ]. The use of mice was approved by the Texas Tech University institutional animal care committee.

### Generation of mouse mAbs specific to the RON PSI domain

Synthetic peptides containing 42 amino acids from Gly^526^ to Pro^568^ corresponding to the RON PSI domain [7] was conjugated to keyhole limpet hemocyanin and used as immunogens for Balb/c mice. After immunization, mice also were injected with NIH3T3-RON cells to boost immune response. Culture supernatants from individual hybridoma cell lines were screened for anti-RON reactivity. IgG antibodies were purified using protein G Sepharose columns as previously described [23].

### Assays for detecting mAbs specific to the RON PSI domain

Purified RON proteins and its various isoforms including short-form RON (sf-RON), RONΔ160, RONΔ110, and RONΔ75 were used as previous described [19]. Purified human MET proteins containing the entire extracellular sequences were from Sino Biologicals [www.sinobiological.com]. A direct enzyme-linked immunosorbent assay (ELISA) was used first to select mAbs specific to the RON PSI domain. Briefly, proteins at 1.5 μg per ml were coated in triplicate in a 96-well ELISA plate followed by addition of individual mAbs at 2 μg/ml. Goat anti-mouse IgG antibody coupled with horseradish peroxidase (HRP) was used as the detecting antibody. The reaction was measured with an ELISA reader. The positivity of mouse mAbs such as PCM5B14 to the RON PSI domain was further evaluated by immunoprecipitation and immunofluorescence analysis.

### Humanization of mouse PCM5B14 and generation of antibody-drug conjugators

Antibody humanization was performed by grafting sequences from complementarity-determining regions (CDRs) of the light and heavy chains of PCM5B14 into human IgG1/κ acceptor frameworks to generate five light chains and five heavy chains, which results in 25 different pairings of humanized PCM5B14 molecules [6, 34]. The subclone H2L4 (designated as H5B14) was selected as the lead candidate. MMAE and DCM linked to a synthetic dipeptide liker (MC-VC-PAB0 from Concortis [www.concortis.com] were used for conjugation with H5B14 according to the manufacturer’s instruction. Conjugation resulted in two H5B14-based ADCs: H5B14-MMAE and H5B14-DCM. H5B14 conjugated with DM1 (H5B14-DM1) and Zt/g4 conjugated with MMAE (Zt/g4-MMAE) also were prepared [1–3]. Control mouse IgG conjugated with MMAE (CmIgG-MMAE) served as the control. All conjugates were verified by hydrophobic interaction chromatography (HIC) for the drug to antibody ratio (DAR). All ADCs were sterilized through a filter and stored at 4 °C for further analysis.

### Assays for RON expression, internalization, and cell viability

Expression of RON by cancer cell lines was determined by flow cytometric analysis using anti-RON mAb Zt/f2 [1–3]. Induction of RON internalization by H5B14 and other anti-RON mAbs was determined by immuno-fluorescence analysis as previously described [1–3]. The internalization efficacy (IE_50_), defined as the time required for a 50% reduction of cell surface RON, was calculated as previously described [1–3]. Cell viability after individual ADC treatment was determined by the 3-[4, 5- dimethylthiazolyl-2]-2, 5diphenyltetrazolium bromide (MTT) assay. Cell death was validated by the trypan blue exclusion assay.

### Analysis of H5B14-based ADC stability in buffer and in human plasma

H5B14-based ADCs at 10 μg per ml were incubated in phosphate-buffered saline (PBS) at room temperature for 28 days. Samples were collected at different time intervals and measured by HIC for changes in DARs. H5B14-based ADCs at 10 μg per ml also were incubated in 1 ml fresh human plasma at 37 °C for 10 days and then collected at different time points. Free MMAE and DCM were measured by using a liquid chromatography with tandem mass spectrometry method (LC-MS/MS) [35] with slight modifications [6].

### Generation of spheroids, isolation of cancer stem-like cells, and quantitation of cells expressing stem cell marker

The spheroid formation derived from ASPC1, BxPc-3, and L3.6pl cell lines with or without H5B14-based ADC treatment was performed as previously described [5, 29]. PAC stem-like cells with RON^+^/CD44^+^/epithelial specific antigen [ESA]^+^ phenotypes (designated as PAC^SL^ cells) were isolated from ASPC1, BxPC-3, and L3.6pl cell lines as previously described [5, 29]. For cell death, PAC^SL^ cells were treated with H5B14-based ADCs for 72 h followed by the trypan blue exclusion assay. Flow cytometric analysis of FG cells expressing stem cell marker aldehyde dehydrogenase (ALDH) after H5B14-based ADC treatment was performed by using ALDEFLUOR™ Kit (Stemcell Technologies, Cambridge, MA).

### Tumor xenograft model and H5B14-based ADC treatment

LoVo, H358, HT-29, L3.6pl, and T-47D cell lines were used in tumor xenograft model. Female athymic nude mice (ten mice per group) were injected with 5 × 10^6^ cells in 0.1 ml PBS into the subcutaneous space of the right flank as previously described [1–3]. Xenograft tumors mediated by LoVo cells served as the negative control. Mice were randomized into control and ADC-treatment groups (five animals per group). Treatment began when tumors had a mean volume of ~ 150 mm^3^. A single dose of H5B14-MMAE or H5B14-DCM at 20 mg/kg was injected through tail vein in a volume of 0.1 ml PBS. Tumor volumes were measured every 4 days and monitored up to 36 days. All mice were sacrificed at the end of study. Tumors from individual mice were collected and weighed to reach an average value for each group. The percentage of inhibition was calculated as previously described [1–3].

### Maximal tolerated doses of H5B14-based ADCs in mice

Female athymic nude mice (five mice per group) were administrated with H5B14-based ADCs at 40, 60, 80, and 100 mg/kg in a single dose through the tail vein. Animals were monitored daily for activity, responsiveness, food consumption, bodyweight, and others. Mice were sacrificed at the end of the study.

### Statistical analysis

The GraphPad Prism 7 software was used for statistical analysis. Results are shown as mean ± SD. The data between control and experimental groups were compared using Student *t* test. Statistical differences at *p* < 0.05 were considered significant.

## Results

### Humanization and characterization of H5B14 specific to the RON PSI domain

**Fig. 1:**
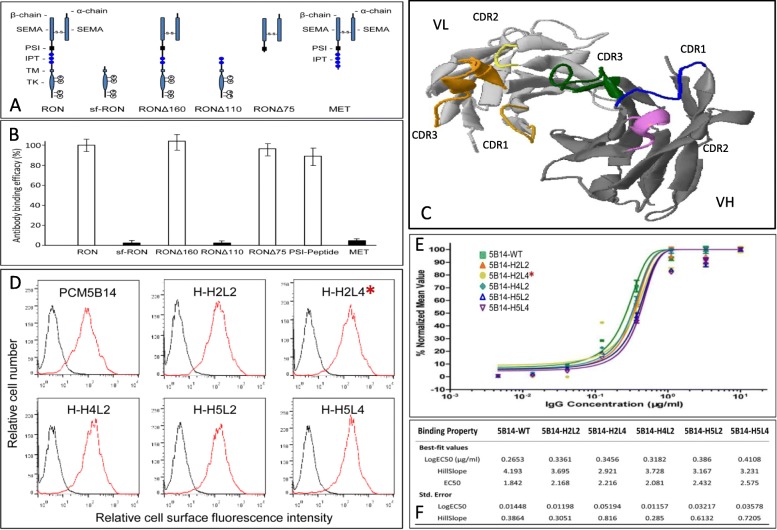
Characterization of mouse mAb specific to the RON PSI domain and its humanization: **a** Schematic representation of RON, RON isoforms, and MET extracellular structure. Mature RON contains a 35 kDa α-chain and a 145 kDa β-chain linked by a disulfide bond. SEMA, PSI and IPT domains are located in the β-chain. The listed four RON isoforms are shown with unique truncations and deletions either by alternative initiation or by mRNA splicing [8]. The MET extracellular structure is similar that of RON. **b** Direct ELISA for PCM5B14 reactive to the RON PSI domain. RON, RON isoforms, RON PSI peptides, and MET extracellular protein were coated at 150 ng per well in triplicate in a 96-well plate. Goat-anti-mouse IgG coupled with HRP was used as the detecting antibody. Results are shown as the percentage of antibody-binding activity. PCM5B14 reactive to RON was set as 100%. **c** Modeling of CDRs from PCM5B14 in human IgG heavy chain and light chain. The framework of human IgG1 molecule was used for PCM5B14 humanization. The models of PCM5B14 CDRs grafted in the variable regions of human IgG1 heavy chain and light chain were generated using the software Automatic Predictions of Immunoglobulin Structures (Tramontano at University of Rome, Italy). **d** Binding of humanized antibody subclones to RON expressed by HT-29 cells. Humanized IgG subclones at 1.5 μg per ml were incubated with HT-29 cells followed by addition of goat anti-human IgG1 antibody coupled with FITC. Immunofluorescence intensity from individual samples was determined by flow cytometric analysis. **e** and **f** Analysis of binding affinities of humanized IgG subclones to human RON. Different amounts of humanized PCM5B14 subclones were incubated with NIH-3 T3 cells expressing human RON followed by addition of goat anti-human IgG1 antibody coupled with fluorescein isothiocyanate (FITC). Antibody-binding affinity was calculated as previously described [6]

Procedures to produce mouse mAb PCM5B14 specific to the RON PSI domain is illustrated in Additional file 1: Figure S1. Using RON, various RON isoforms, and the MET extracellular protein (Fig. 1a) as antigens in the ELISA assay, we confirmed that PCM5B14 is specific to the RON PSI domain but not to MET (Fig. 1b). Composition of amino acids from individual CDRs of PCM5B14 were obtained by sequence analysis. Schematic structures of CDRs from PCM5B14 grafted into both light and heavy chains of human IgG1/κ acceptor frameworks are shown in Fig. 1c. A total of 25 pairs of humanized IgG1/κ molecules were obtained from five light chains in combination with five heavy chains. Immunofluorescence analyses of individual humanized antibodies binding to cell surface RON in HT-29 cells in comparison with mouse PCM5B14 are shown in Fig. 1d. Analysis of antibody-binding affinities by individual humanized antibodies is presented in Fig. 1e and f**.** H5B14, the humanized IgG1/κ subclone H2L4 with the binding affinity of 0.35 μg per ml, was selected for further study. Large scale production of H5B14 up to 150 mg IgG protein was achieved using HEK293 expression system [1–3]. H5B14 also recognized cynomolgus monkey RON with a binding affinity of 0.41 μg/ml but not mouse RON (Additional file 2: Figure S2**)**.

### Superiority of H5B14 in induction of RON internalization for drug delivery

**Fig. 2:**
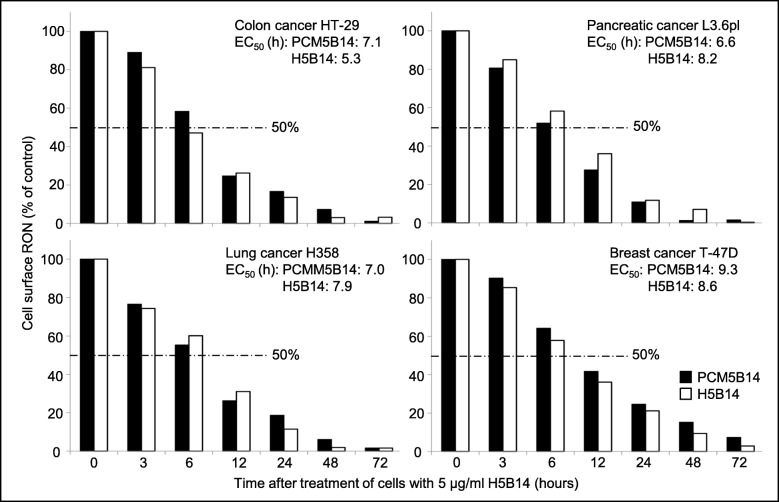
H5B14-induced cell surface RON internalization: Four cancer cell lines HT-29, L3.6pl, H358, and T-47D at 1 × 10^6^ cells per dish were treated at 37 °C with 5 μg/ml of H5B14 or PCM5B14, collected at different time points, washed with acidic buffer to eliminate cell surface bound IgG [1], and then incubated with 2 μg/mL of anti-RON mAb Zt/c1 [23]. Immunofluorescence was analyzed by flow cytometer using FITC-coupled anti-mouse IgG. Immunofluorescence from cells treated with H5B14 at 4 °C was set as 100%. Internalization efficiency (IC_50_) was calculated as the time required to achieve a 50% reduction of cell surface RON

H358, HT-29, L3.6pl, and T-47D cell lines expressing variable levels of RON [1–6] were used to evaluate the effectiveness of H5B14 in induction of RON internalization (Fig. 2). The calculated IE_50_ values were 5.3 h for HT-29; 8.2 h for L3.6pl; 7.9 h for H358; and 8.6 h for T-47D cells, respectively. Similar results were also observed when PCM5B14 was used (Fig. 2). These results suggest that H5B14 has the ability to cause a rapid RON internalization with more than 50% RON internalized within a 10 h period.

We then determined whether H5B14-induced RON internalization is superior over other anti-RON mAbs that recognize the SEMA domain or the IPT motifs. Results for such comparisons were shown in Additional file 6: Table S1. Among eight RON-expressing cell lines tested, average IE_50_ values were 8.66 h ± 2.36 for PCM5B14 and 8.77 h ± 2.39 for H5B14, respectively. In contrast, average IE_50_ values for Zt/g4 and Zt/f12 that recognize the sema domain were 14.90 h ± 6.23 and 17.12 h ± 3.62, respectively. The effect of Zt/c1, which recognizes the IPT motifs, appeared to be weak with an average IE_50_ value at 19.90 h ± 3.83. Statistical analysis confirmed that IE_50_ from H5B14 is significantly lower than those from other mAbs (Additional file 6: Table S1). Thus, H5B14 binding to the RON PSI domain is superior over other anti-RON mAbs in induction of RON internalization.

### H5B14-based ADCs and their stability in human plasma

**Fig. 3:**
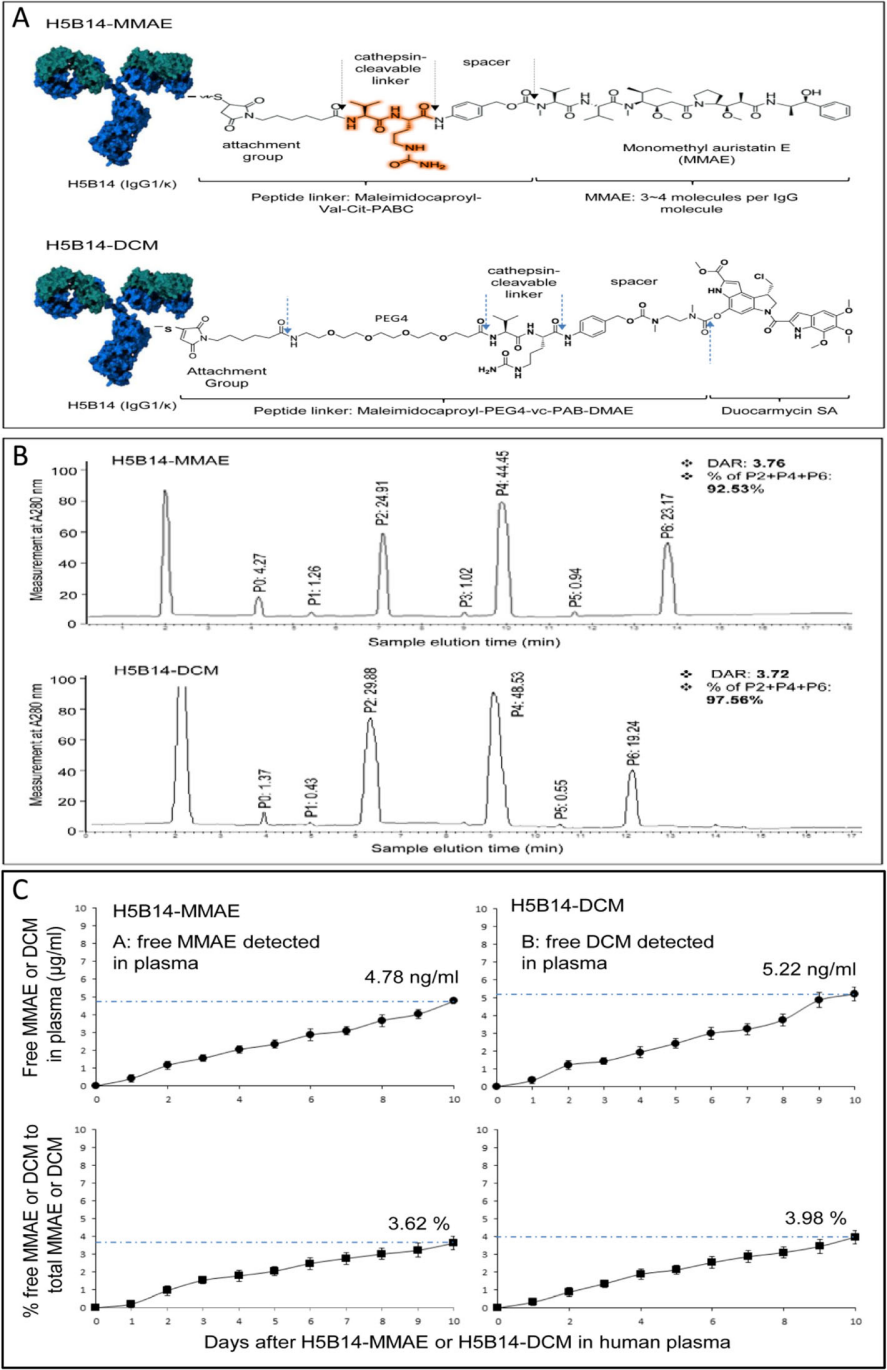
Schematic Structures of H5B14-based ADCs, drug conjugation profiles, and their plasma stability: **a** Schematic representation of H5B14-based ADCs. Both MMAE and duocarmycin [DCM] were conjugated to H5B14 by the valine-citruline dipeptide linker according to the manufacturer’s instruction (www.concortis.com). **b** HIC analysis of MMAE and DCM conjugated to H5B14. Individual H5B15-MMAEs or H5B14-DCMs with different numbers of MMAE or DCM [0–6] are marked as P0 to P6. The DAR combining P2, P4, and P6 was calculated at 3.76:1 for H5B14-MMAE and 3.73:1 for H5B14-DCM. **c** Dissociation MMAE or DCM from H5B14-based ADCs in human plasma. Both H5B14-MMAE and H5B14-DCM at 10 μg per ml were incubated with fresh human plasma at 37 °C for 10 days. The amount of free MMAE or DCM in plasma was determined using the LC-MS/MS method [35] with slight modifications [6]. Samples also were used for measuring MMAE or DCM conjugated H5B14 as detailed in Materials and Methods. A ratio from free MMAE to the total MMAE in H-Zt/g4-MMAE was calculated to determine the percentage of MMAE dissociated from H-Zt/g4-MMAE

Schematic representatives of H5B14-MMAE and H5B14-DCM are shown in Fig. 3a. Conjugation profiles of both H5B14-MMAE and H5B14-DCM (Fig. 3b) fitted those of ADCs formulated using the dipeptide linker as previously described [4, 5]. Average DARs were 3.76:1 for H5B14-MMAE and 3.72 for H5B14-DCM 3.72:1 (Fig. 3b). Stability analysis of H5B14-MMAE and H5B14-DCM in PBS at room temperature up to 28 days showed that both ADCs are highly stable (Additional file 3: Figure S3). The obtained DAR at day 28 was 3.59 for H5B14-MMAE and 3.48 for H5B14-DCM. Both ADCs also were stable in human plasma with less than 6% of MMAE or DCM dissociated from the conjugates after incubation at 37 °C for 10 days (Fig. 3c). These results indicate that H5B14-based ADCs are highly stable in PBS and in human plasma with minimal dissociation.

### Effect of H5B14-based ADCs on cancer cell viability

**Fig. 4:**
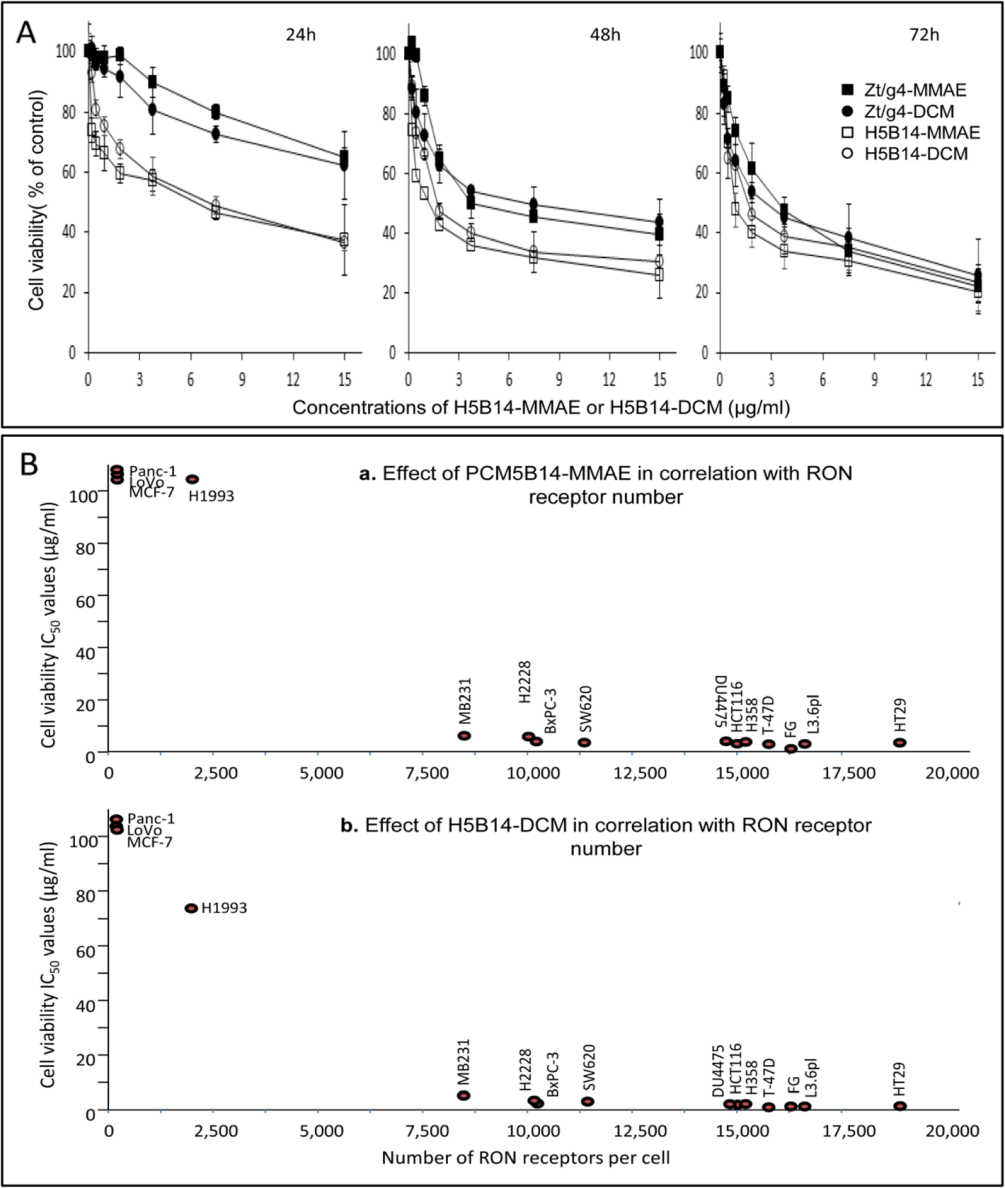
Efficacy of H5B14-based ADCS in vitro on cell viability and its association with levels of RON expression: **a** Effect of H5B14-based ADCs on cancer cell viability. H358 cells (8000 cells per well in a 96-well plate in triplicate) were treated with different amounts of H5B14-MMAE or H5B14-DCM for 72 h. Cell viability was determined by the MTT assay. **b** Correlation between levels of RON expression and efficacy of H5B14-based ADCs. Cell viability IC_50_ values from a panel of 15 cancer cell lines expressing variable levels of RON were plotted with different numbers of RON expressed per cell. H5B14-based ADCs at the amount below 5 μg per ml to achieve an IC_50_ value was used as the effective dose to determine the required receptor number to reach the EC_95_ value. The IC_50_ values for cell viability from individual groups were calculated using the GraphPad Prism 7 software. Results shown here are from one of two experiments with similar results

Because H5B14 induces a robust RON internalization, we wanted to know the impact of H5B14-MMAE and H5B14-DCM on cancer cell viability. A time dependent study showed that both H5B14-MMAE and H5B14-DCM decrease H358 cell viability at relatively early stages (Fig. 4a). As judged by the dose of 3.75 μg/ml, H5B14-based ADCs progressively reduced cell viabilities from 100% to ~ 60% and to ~ 40% within 24 h to 48 h, respectively. In contrast, cell viabilities treated with the same dose of Zt/g4-based ADCs remained at ~ 90% and ~ 55%, respectively. These studies indicate that H5B14-mediated drug delivery significantly reduces cell viability at the early stages after ADC treatment.

We then studied H5B14-mediated drug delivery in association with its efficacy. A panel of 15 cancer cell lines with variable RON expression was tested. Zt/g4-based ADCs served for comparison. H5B14-MMAE in a dose-dependent manner significantly reduced cell viability in all RON-positive cell lines tested (Additional file 4: Figure S4A). Similar results were also obtained when H5B14-DCM was used (Additional file 4: Figure S4B). A summary of H5B14-based ADCs in reduction of cell viability with IC_50_ values is presented in Table 1**.** It was noticed that the efficacy between H5B14- and Zt/g4-based ADCs is at comparable levels, although differences in induction of RON internalization existed between H5B14 and Zt/g4 (Additional file 6: Table S1). The average IC_50_ at 72 h was 3.06 ± 1.36 μg/ml for H5B14-MMAE, comparable to 2.95 ± 1.52 μg/ml for Zt/g4-MMAE. Similarly, the average IC_50_ was 2.43 ± 1.22 μg/ml for H5B14-DCM, similar to 2.61 ± 1.36 μg/ml for Zt/g4-DCM. Thus, H5B14-based ADCs is as effective as Zt/g4-based ADCs in reduction of cell viability.

**Table 1:** Inhibitory effect of H5B14-based ADCs on cell viability from a panel of cancer cell lines

Cancel cell lines	IC50 values of H5B14-MMAE and H5B14-DCM in cell viability (μg/ml)^a^					
	Zt/g4-MMAE	Zt/g4-DCM	PCM5B14-MMAE	PCM5B14-DCM	H5B14-MMAE	H5B14-DCM
MCF-7	> 100	> 100	> 100	> 100	> 100	> 100
DU4475	2.28	3.96	2.75	2.46	3.04	1.81
MDA-MB-231	6.89	3.48	5.38	5.98	6.25	5.30
T-47D	1.77	1.22	3.11	1.55	2.35	1.63
LoVo	> 100	> 100	> 100	> 100	> 100	> 100
HCT-116	2.21	0.94	3.31	1.58	2.83	1.37
HT-29	1.65	1.65	3.15	2.97	2.88	2.09
SW-620	2.97	2.65	5.44	1.92	3.35	2.95
H1993	> 100	83.89	> 100	> 100	> 100	74.07
H2228	3.86	4.28	5.63	4.65	4.49	3.64
H358	3.85	2.96	2.77	3.51	1.46	1.87
Panc-1	> 100	> 100	> 100	> 100	> 100	> 100
BxPC-3	3.04	3.89	3.25	5.25	2.68	3.02
FG	2.14	0.74	1.13	0.96	1.34	1.15
L3.6pl	1.83	2.41	2.47	4.48	3.03	1.88
Average	2.95 ± 1.52	2.61 ± 1.36	3.49 ± 1.41	3.21 ± 1.68	3.06 ± 1.36	2.43 ± 1.22

^a^individual cancer cell lines at ~ 8000 cells per well were cultured in triplicate in the presence of absence of different amounts of H5B14-MMAE or H5B14-DCM for 72 h. Zt/g4-based ADCs and PCM5B14-based ADCS were used for comparison. The MTS assay was used to determine the cell viability. The IC50 values for individual cell lines were calculated using the GraphPad Prism 7 software as previously described [1]

The relationship between the effect of H5B14-based ADCs on cell viability and the number of RON receptors expressed by cancer cells also was analyzed (Fig. 4b). A direct correlation was established by plotting the individual IC_50_ values against the cell surface number of RON. Although sensitivities of individual cell lines to ADCs were different, patterns of their responsiveness to H5B14-MMAE or H5B14-DCM were highly similar. We reasoned that the minimal number of cell-surface RON required for H5B14-based ADCs to achieve a 95% reduction in cell viability is ~ 8000 molecules per cell. A decrease in the number of RON molecules correlate proportionally with the diminished efficacy of H5B14-based ADCs. Thus, cancer cells expressing ~ 8000 RON molecules per cell are in vitro required for H5B14-based ADCs to show maximal activities.

### Inhibitory effect of H5B14-based ADCs on spheroid formation and survival of cancer stem-like cells

**Fig. 5:**
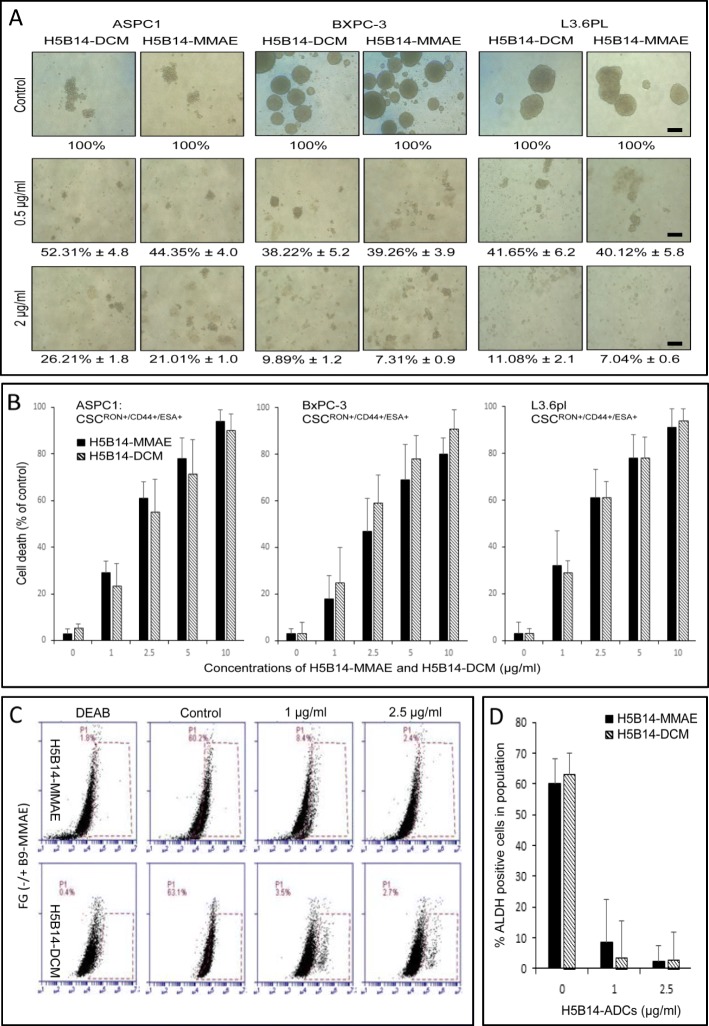
Effect of H5B14-based ADCs on cancer stem cell-derived spheroid formation, cellular viability, and ALDH expression. **a** Inhibitory effect of H5B14-based ADCs on spheroid formation by pancreatic cancer cells. Spheroid formation from ASPC-1, BxPC3, and L3.6pl cells were performed as previously described [5, 29]. H5B14-MMAE or H5B14-DCM was added after initiation of cell culture. The number of spheroids was counted 40 days after ADC treatment. Scale bar: 50 μM. **b** Death of pancreatic stem-like cells mediated by H5B14-based ADCs. PAC^SL^ cells with RON^+^/CD44^+^/ESA^+^ phenotypes were treated in triplicate with different amounts of H5B14-MMAE or H5B14-DCM for 72 h. Cell death was determined by the trypan blue exclusion assay [1]. **c** and **d** Inhibitory effect of H5B14-based ADCs on pancreatic cancer cells expressing ALDH. FG cells expressing a relatively high level of ALDH were used as the model. After treatment of cells with H5B14-MMAE or H5B14-DCM for 48 h, the percentages of FG cells expressing ALDH were determined by using the ALDEFLUOR™ Kit according to the manufacture’s instruction. Results shown here are from one of two experiments with similar results

To determine H5B14-based ADCs in cancer stem-like cells, we first studied the effect of H5B14-MMAE and H5B14-DCM on spheroid formation using ASPC1, BxPc-3, and L3.6pl cell lines as the model. Treatment of individual cell lines with H5B14-based ADCs dramatically attenuated the ability of these cells to form spheroids (Fig. 5a). For example, the number of spheroids derived from BxPC-3 cells was significantly reduced. Similar results were also observed for spheroids formed by ASPC1 and L3.6pl cell lines (Fig. 5a). Thus, H5B14-based ADCs have the ability to impair the spheroid formation mediated by PAC cells.

We then studied H5B14-based ADCs in induction of PAC^SL^ cell death. PAC^SL^ cells expressing RON, CD44, and ESA are known to be tumor-initiating stem-like cells [36, 37]. H5B14-MMAE or H5B14-DCM treatment caused PAC^SL^ cell death in a dose-dependent manner (Fig. 5b). Among three PAC^SLC^ cell lines tested, the IC_50_ values were in the range of ~ 2 μg per ml for both H5B14-based ADCs, indicating that H5B14-based ADCs in vitro is capable of killing PAC^SL^ cells.

Finally, we determined the effect of H5B14-based ADCs on FG cells expressing ALDH. Percentages of FG cells expressed ALDH were at relatively high levels [~ 60% of cells positive] (Fig. 5c and d). However, percentages of ALDH-positive cells were dramatically decreased to 8.4% in H5B14-MMAE treated cells and to 3.5% in H5B14-DCM treated cells. These results suggest that H5B14-based ADCs are effective in decreasing FG cells expressing ALDH.

### Therapeutic efficacy of H5B14-based ADCs in multiple tumor xenograft models

**Fig. 6:**
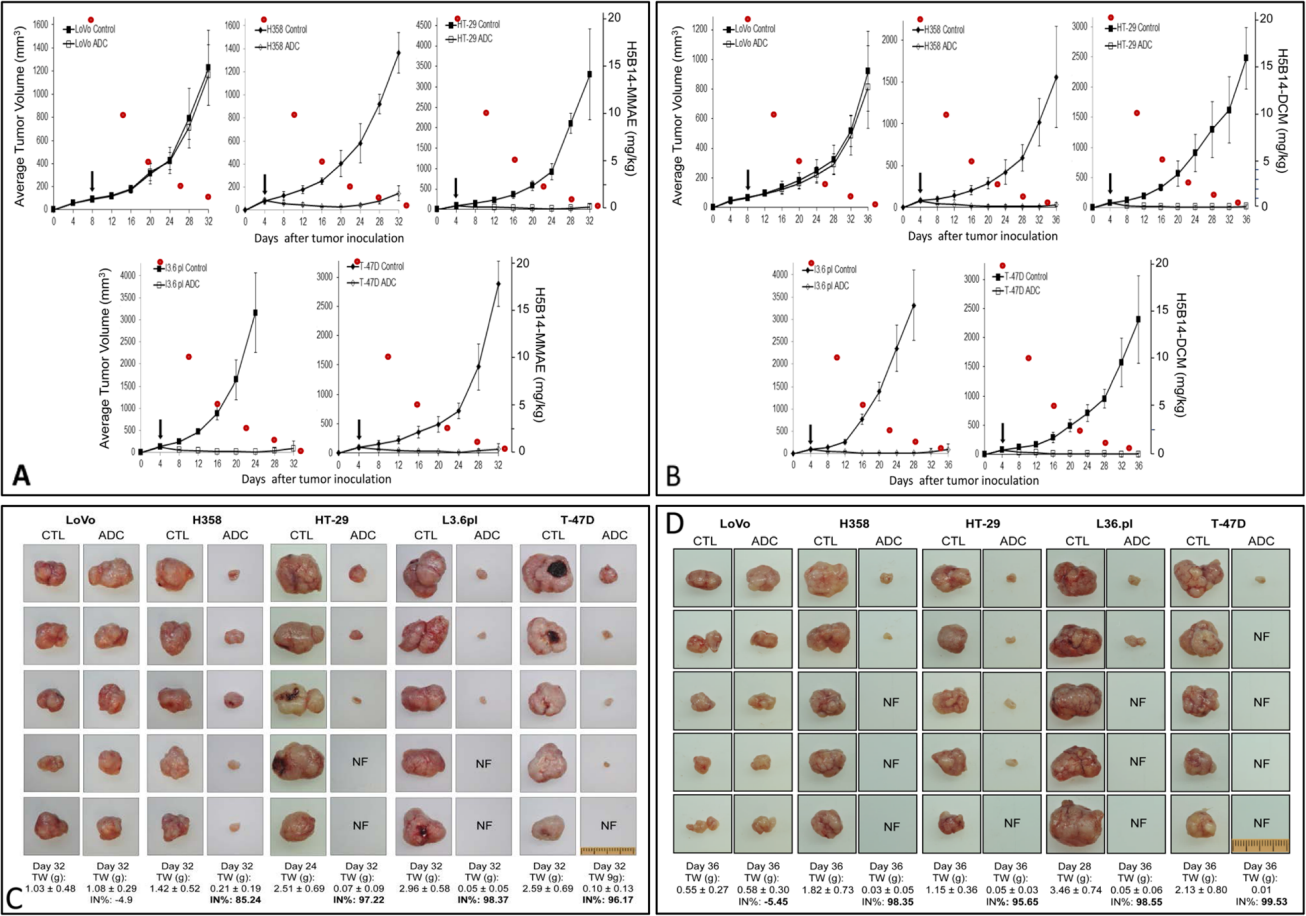
Therapeutic efficacy of H5B14-based ADCs in multiple tumor xenograft models: **a** and **b** Inhibitory effect of H5B14-MMAE and H5B14-DCM on xenograft tumor growth. Tumors mediated by H358, HT-29, L3.6pl, and T-47D cell lines were used as the model. LoVo cell-derived tumors without RON expression served as the control. Athymic nude mice (5 mice per group) were subcutaneously inoculated with 5 × 10^6^ cells. H5B14-MMAE (**a**) or H5B14-DCM (**b**) at 20 mg/kg in a single injection was administered through tail vein after tumors volumes reached ~ 150 mm^3^. Mice injected with CmIgG-MMAE at 20 mg/kg were used as the control. To establish the effect-time relationship, the estimated reduction of H-Zt/g4-MMAE in vivo according to the t½ was marked as red circles [6]. **c** and **d** Effect of H5B14-based ADCS on tumor weight and number. Individual tumors from different groups described in (**a**) and (**b**) were collected at the end of study. All tumors were weighed to reach the average tumor weight per group, which was used to obtain the percentages of tumor growth inhibition. The number of tumors was counted to determine the eradication effect of H5B14-based ADCs. NF, no tumors were found in the injected site

Both H5B14-MMAE and H5B14-DCM at 20 mg/kg in a single injection regimen were evaluated in mouse xenograft tumor models initiated by H358, HT-29, L3.6pl, and T-47D cell lines. LoVo cells served as the control. Injection of H5B14-MMAE inhibited the growth of xenograft tumors mediated by all four cell lines, respectively (Fig. 6a). The effect of H5B14-MMAE on H358 cell-mediated tumor growth appeared to be relatively weak (Fig. 6a). In contrast, H5B14-DCM inhibited tumor growth-mediated by all four cancer cell lines without visible differences (Fig. 6b). Considering the terminal half-life [*t*½: ~ 6.3 days] of humanized anti-RON ADCs in mice [1–6] and the tumor regrowth curve, we calculated tumoristatic concentrations [TSCs, the minimal concentrations required to balance tumor growth and inhibition]. TSCs for H5B14-MMAE treated animals were in the range of 0.63 mg per kg for tumors mediated by HT-29, L3.6pl, and T-47D cells, respectively (Fig. 6a). TSCs for H358 xenograft tumors were in the range of ~ 1.25 mg per kg. For H5B14-DCM treated mice, TSCs for all four tumor xenograft models were in the range of ~ 0.63 mg/kg (Fig. 6b). The tumor regrowth appears to be at day 30 for both H5B14-based ADCs. The only exception was H358 xenograft tumors treated with H5B14-MMAE, in which tumor regrowth was observed at day 24. Regardless these differences, results in Fig. 6a and b demonstrate that H5B14-based ADCs at a single injection is highly effective and its activity is long lasting.

We further compared the tumor number and weight at the end of study. H5B14-MMAE reduced the average tumor weight by 85.24% for H358, 97.22% for HT-29, 98.37% for L3.6pl, and 96.17% for T-47D xenograft tumors, respectively (Fig. 6c). Similarly, the average decrease in tumor weight caused by H5B14-DCM was 98.35% for H358, 95.65% for HT-29, 98.55% for L3.6pl, and 99.53 for T-47D xenograft tumors, respectively (Fig. 6d). It is worthy to note that although tumors from H5B14-MMAE treated L3.6pl group were collected 8 days later [Day 24 for control mice versus day 32 for experimental group], a significant decrease in the average tumor weight was still observed (Fig. 6c). These results demonstrate that H5B14-based ADCs inhibits tumor growth leading to reduced tumor weight.

Both H5B14-MMAE and H5B14-DCM showed tumor-eradicating activities (Fig. 6c and d). By counting the number of tumors eliminated from both ADC-treated mice, the effect of H5B14-DCM is slightly stronger than that of H5B14-MMAE. As noticed, H5B14-MMAE did not eradicate any tumors from the H358 xenograft model (Fig. 6c). In contrast, H5B14-DCM eliminated three tumors from the H358 tumor model (Fig. 6d). These results indicate that both H5B14-MMAE and H5B14-DCM at 20 mg/kg in a single injection are capable of eradicating tumor xenografts dependent on individual cell lines used.

### Toxicological effect of H5B14-based ADCs in mouse bodyweight

A single injection of H5B14-based ADCs at 40, 60, 80, and 100 mg/kg was used to determine the maximum tolerated dose in mice (Additional file 5: Figure S5). Mice receiving both H5B14-MMAE and H5B14-DCM up to 60 mg/kg within 12 days displayed the normal daily activity, food consumption, and bodyweight. However, both H5B14-MMAE and H5B14-DCM at 80 mg/kg caused a dramatic bodyweight reduction. Furthermore, death [three out of five mice] occurred in mice receiving 100 mg/kg H5B14-MMAE or H5B14-DCM. These studies imply that H5B14-based ADCs below 60 mg/kg is relatively safe in mice as judged by daily activity, food consumption, and bodyweight.

## Discussion

The study presented here describes humanized antibody H5B14 specific to the RON PSI domain and its efficacy in the form of ADC for targeted cancer therapy. First, we generated H5B14 from mouse PCM5B14, which is highly specific to the RON PSI domain and confirmed that the binding of H5B14 to the PSI domain causes robust RON internalization, leading to effective drug delivery. Second, we demonstrated that H5B14-based ADCs are highly stable in human plasma with minimal dissociation within a prolonged period, which could have a favorable pharmacokinetic profile in vivo. Third, we confirmed that H5B14-based ADCs affect cancer cell viability in the early stages, inhibit spheroid formation, and cause cancer stem-like cell death. Fourth, we confirmed that a single injection of H5B14-based ADCs not only inhibits but also eradicates tumors in multiple xenograft models. Finally, we demonstrate that both H5B14-MMAE and H5B14-DCM are well tolerated up to 60 mg/kg in mice. Thus, H5B14-based ADCs are superior with tumor-eradicating activity, which warrants for clinical trials in the future.

ADC-mediated receptor internalization followed by intracellular drug processing is a critical step required for cancer cell killing [38–40]. Several strategies such as the use of mAbs recognizing distinct epitopes have been used to accelerate receptor internalization [40]. Also, a bispecific antibody targeting the lysosomal antigen has been applied to improve the payload delivery [41]. We have previously demonstrated that anti-RON mAb Zt/g4 specific to the RON SEMA domain causes RON internalization [1–6], which mimics the ligand-induced RON endocytosis [31, 32]. Nevertheless, the action of Zt/g4 is moderated with an average internalization efficacy at 15 h [1–6]. To improve antibody-mediated intracellular drug delivery, we focused on mAbs targeting the PSI domain due to its unique location and function in the RON extracellular sequence [7, 8]. The use of antibody specific to the PSI domain for induction of MET internalization has been reported [33]. However, the use of mAbs targeting the PSI domain for drug delivery has not been studied. By generating H5B14 from mouse PCM5B14, we demonstrated that H5B14 specific to the RON PSI domain rapidly induces robust RON internalization. Among three extracellular domains (SEMA, PSI and IPT) evaluated, H5B14 binding to the RON PSI domain appears to be most effective in induction of RON internalization with the average internalization efficacy at ~ 9 h. This is a significant improvement in comparison with other anti-RON mAbs recognizing the SEMA domain or the IPT motifs. Thus, the binding of antibodies to different regions in the RON extracellular sequences has an impact on RON internalization. Nevertheless, RON internalization is affected by other factors such as levels of RON expression. As shown in Fig. 4b, H5B14-mediated RON internalization is propositionally correlated with the number of RON molecules on the cell surface. Specifically, H5B14-mediated internalization appears to be more effective in cells expressing high number of RON in the cell surface.

The beneficial effect from increased RON internalization is evident in functional study. As shown in Fig. 4a, the inhibitory effect of H5B14-MMAE or H5B14-DCM on cell viability [more than 60% decrease] was observed as early as 24 h after cells were exposed to ADCs. Both H5B14-MMAE and H5B14-DCM act equally. This effect is not observed in cells treated with Zt/g4-based ADCs, which show the effect at relatively later stages due to its moderate internalization activity. For example, the significant reduction of cell viability caused by Zt/g4-based ADCs [more than 60% reduction] was only documented 72 h after drug treatment. Thus, H5B14-mediated rapid RON internalization leads to effective drug delivery and therefore has the pharmacological advances in controlling cancer cell growth and survival. Such activity could have a potential clinical implication.

Functional studies also demonstrate the inhibitory and/or cytotoxic effects of H5B14-Based ADCs on cancer stem-like cells. As a biomarker, RON expression is sustained in cancer stem-like cells including those from pancreatic and breast cancer cells [5, 6, 29]. Targeting RON using mAbs or tyrosine kinase inhibitors has been shown to eradicate cancer stem-like cells [5, 6, 29]. We showed here that H5B14-based ADCs are effective in inhibition of spheroid formation derived from PAC stem-like cells. Also, these ADCs are capable of causing death of PAC^SL^ cells with RON^+^/CD44^+^/ESA^+^ phenotypes. PAC^SL^ cells are known as tumor-initiating cells capable of self-renewal and pluripotency [36, 37]. We further confirmed that H5B14-based ADCs are capable of decreasing the percentages of FG cells expressing ALDH, a reliable cancer stem cell marker for solid tumors [42]. These findings highlight the importance of potential use of H5B14-based ADCs for targeting cancer stem-like cells to inhibit and/or to eradicate tumor-initiating cells in vivo.

The efficacy of H5B14-based ADCs in vivo was confirmed in multiple tumor xenograft models. The reasons to select H358, HT-29, L3.6pl, and T-47D cell lines for generating xenograft tumors were based on their tissue originality, malignant status, RON expression level, and responsiveness in vitro to anti-RON ADCs. Moreover, the rate of tumor growth in vivo is different among them. For example, L3.6pl cell-mediated tumors grew extremely fast. In contrast, tumor growth mediated by H358 cells was relatively slow. Consideration of these facts helped us to objectively evaluate the efficacy of H5B14-based ADCs in vivo. The rationale of using H5B14-based ADCs at 20 mg/kg in a single injection as the treatment regimen was to determine the relationship between the effective doses of ADCs and the duration of their activity. Humanized ADCs conjugated either with MMAE or DM1 has a *t*½ of ~ 6.3 days in mouse [1–6]. Thus, the use of a relatively high dose in a single injection regimen should help establish a dose-dependent efficacy in correlation with the *t*½ of ADCs.

Results presented in Fig. 6 confirm that both H5B14-MMAE and H5B14-DCM are effective in inhibition and/or eradication of multiple tumor xenografts. Administration of H5B14-MMAE or H5B14-DCM immediately prevented xenograft tumor growth and caused a continued reduction in tumor volume in all models tested. Both ADCs are capable of inhibiting tumor growth regardless their origination or rate of tumor growth. Although differences existed between two ADCs in inhibition of tumor growth, both ADCs show a long-lasting effect as evident by the lack of tumor regrowth at day 32 for H5B14-MMAE treated mice and at day 36 for H5B14-DCM treated mice. These activities were confirmed after five-cycles of the *t*½ of ADCs. One exception is H358 xenograft tumors treated with H5B14-MMAE, in which tumor regrowth was observed at day 28. By analyzing curves of tumor growth, it appears that the efficacy of H5B14-MMAE is slight weak in comparison with that of H5B14-DCM. This notion was supported by measuring the average tumor weight and by accounting the number of tumors eradicated from both ADC-treated animals. As shown in Fig. 6c and d, tumor eradication was observed in all xenograft models treated with H5B14-DCM with a total of 11 tumors eliminated. However, H5B14-MMAE-mediated tumor eradication was observed only in three tumor xenograft models with a total of five tumors eliminated. No eradication was observed in H358 cell-mediated tumors. Considering these facts, we believe that the use of a relatively high dose of H5B14-based ADCs in the initial phase to inhibit and eradicate tumor xenografts could be an interesting strategy for RON-targeted cancer therapy.

## Conclusions

ADCs targeting the RON receptor with significantly improved therapeutic index are the key for the success in cancer therapy. Currently, antibody-based biotherapeutics specific to RON are under intensive evaluation in both preclinical models and clinical trials. The results shown here indicate that humanized anti-RON mAb H5B14 targeting the RON PSI domain, leading to increased cytotoxic drug delivery and effective inhibition and/or eradication of multiple xenograft tumors, is an attractive strategy for generation of RON-targeted ADCs for potential clinical application. Our study highlights the importance of selecting anti-RON mAbs with unique features for ADC development and lays the foundation for using H5B14-based ADCS for clinical trials in the future.

## Supplementary information

**Additional file 1: Figure S1.** Schematic representation of procedures for generation of mouse mAbs specific to the RON PSI domain. (A) Structure and sequence of the human RON PSI domain. A peptide containing 43 amino acids from Gly526 to Pro568 corresponding to the entire PSI domain was presented. (B) The synthetic peptide conjugated to KLH was used for mouse immunization. Individual hybridomas cell lines were obtained through classical methods for mouse mAb production. The mAbs specific to the RON PSI domain such as PCM5B14 were verified by ELISA, immunoprecipitation, and immunofluorescence analyses.

**Additional file 2: Figure S2.** Interaction of H5B14 with RONs from different species. Stable NIH3T3 cells expressing human, monkey, or mouse RON were incubated in duplicate with different amounts of H5B14 followed by goat anti-human IgG coupled with FITC. Immunofluorescent intensities from individual samples were determined by flow cytometric analysis. Results are shown as the percentages of H5B14 specific binding to RON. The binding affinity (IC50) was calculated using the GraphPad Prism 7 software.

**Additional file 3: Figure S3.** Stability of H5B14-based ADCs in PBS. H5B14-MMAE and H5B14-DCM at 10 μg/ml were incubated with 1 ml PBS at room temperature for 28 days. Samples were collected at different time intervals and analyzed by HIC. Individual peaks with different numbers of MMAE or DCM conjugated to H5B14 were marked as P0 to P6. The average DAR combining P2, P4, and P6 for both ADCs were calculated accordingly [1–3].

**Additional file 4: Figure S4.** The concentration-dependent effect of H5B14-based ADCs on cell viability. A panel of fifteen cancer cell lines expressing variable levels of RON was used as the model. Cells at 8000 cells per well in a 96-well plate in triplicate were treated with different amounts of H5B14-MMAE (A) or H5B14-DCM (B) for 72 h. Cell viability was determined by the MTT assay. Zt/g4-MMAE or Zt/g4-DCM were used for comparison.

**Additional file 5: Figure S5.** Effect of H5B14-based ADCs on mouse bodyweight. Female athymic nude mice (five mice per group) were injected with H5B14-MMAe or H5B14-DCM at 40, 60, 80, and 100 mg/kg in a single dose through the tail vein, respectively. Animals were monitored daily for activity, responsiveness, food consumption, and others. Individual mice were weighted every day to reach an average bodyweight for each group. All animals were sacrificed at the end of the study.

**Additional file 6: Table S1.** Efficacy of H5B14-Mediated RON Internalization in Comparison with Other Anti-RON mAbs.

## Data Availability

Not applicable.

## Abbreviations

ADC: Antibody-drug conjugates
CDR: Complementarity-determining region
CRC: Colorectal cancer
DAPI: 4′,6-diamidino-2-phenylindole
DAR: Drug to antibody ratio
DM1: Maytansinoid derivative 1
ELISA: Enzyme-linked immunosorbent assay
ESA: Epithelial-specific antigen
FITC: Fluorescein isothiocyanate
HIC: Hydrophobic interaction chromatography
KLH: Keyhole limpet hemocyanin
LC-MS/MS: Liquid chromatography with tandem mass spectrometry
mAb: Monoclonal antibodies
MET: Mesenchymal-epithelial transition
MMAE: Monomethyl auristatin E
MTT: 3-(4, 5- dimethylthiazolyl-2)-2, 5diphenyltetrazolium bromide
PDC: Pancreatic adenocarcinoma
PK: Pharmacokinetic
RON: Recepteur d’origine nantais
