# Supplementary material for: Therapeutic efficacy of a novel humanized antibody-drug conjugate recognizing plexin-semaphorin-integrin domain in the RON receptor for targeted cancer therapy
Source: J Immunother Cancer. 2019 Sep 13;7:250. doi: 10.1186/s40425-019-0732-8 (PMC6743155; doi:10.1186/s40425-019-0732-8)
Supplement: Supplementary file 1 — Additional file 1: Figure S1. Schematic representation of procedures for generation of mouse mAbs specific to the RON PSI domain. (A) Structure and sequence of the human RON PSI domain. A peptide containing 43 amino acids from Gly526 to Pro568 corresponding to the entire PSI domain was presented. (B) The synthetic peptide conjugated to KLH was used for mouse immunization. Individual hybridomas cell lines were obtained through classical methods for mouse mAb production. The mAbs specific to the RON PSI domain such as PCM5B14 were verified by ELISA, immunoprecipitation, and immunofluorescence analyses. [file 40425_2019_732_MOESM1_ESM.pdf]

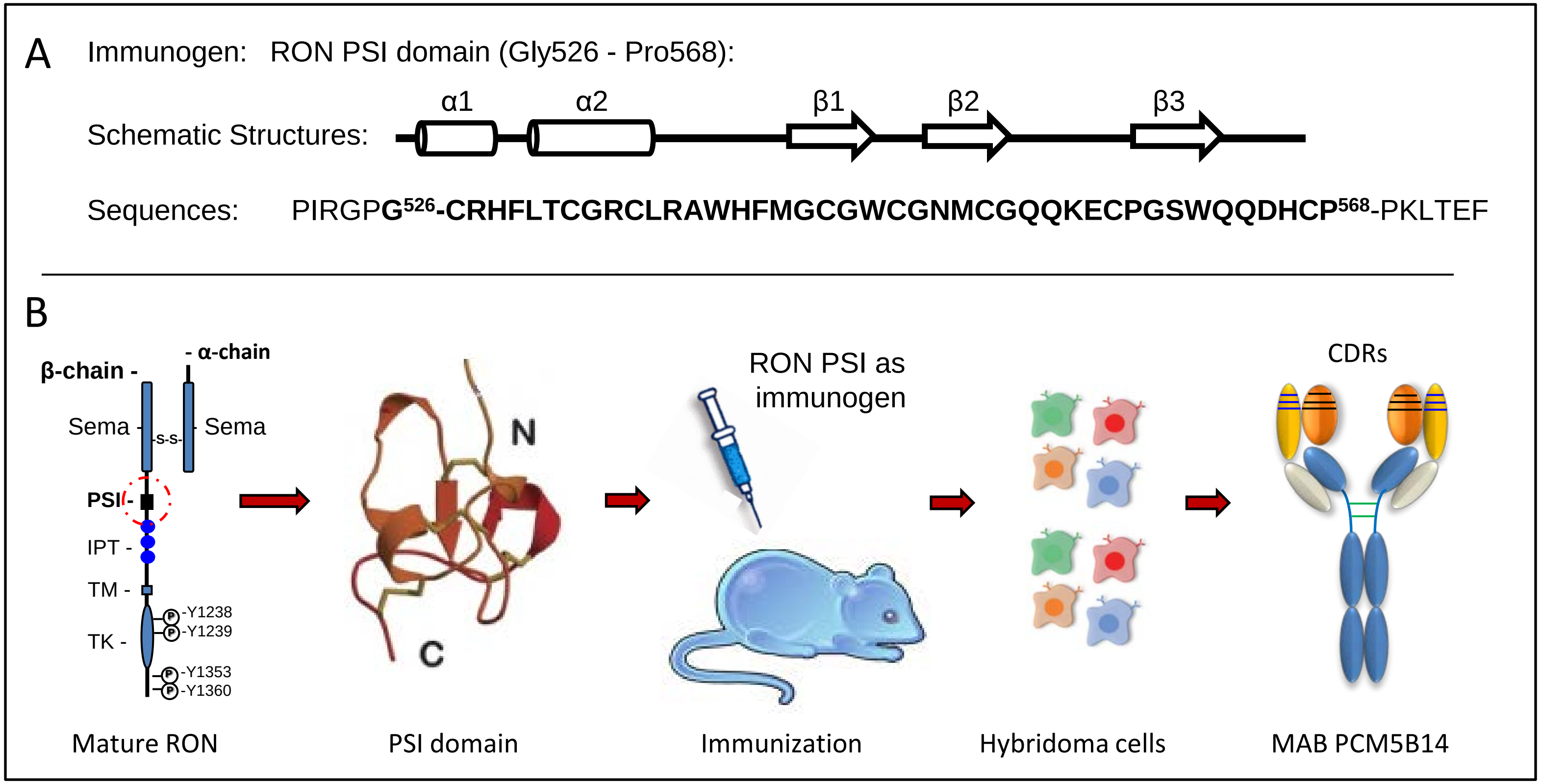

Figure S1 Schematic representation of procedures for generation of mouse mAbs specific to the RON PSI domain. (A) Structure and sequence of the human RON PSI domain. A peptide containing 43 amino acids from Gly^{526} to Pro^{568} corresponding to the entire PSI domain was presented. (B) The synthetic peptide conjugated to KLH was used for mouse immunization. Individual hybridomas cell lines were obtained through classical methods for mouse mAb production. The mAbs specific to the RON PSI domain such as PCM5B14 were verified by ELISA, immunoprecipitation, and immunofluorescence analyses.

Xiang-Min Tong, et al., Supplementary Figure 1
