# Supplementary material for: Therapeutic efficacy of a novel humanized antibody-drug conjugate recognizing plexin-semaphorin-integrin domain in the RON receptor for targeted cancer therapy
Source: J Immunother Cancer. 2019 Sep 13;7:250. doi: 10.1186/s40425-019-0732-8 (PMC6743155; doi:10.1186/s40425-019-0732-8)
Supplement: Supplementary file 2 — Additional file 2: Figure S2. Interaction of H5B14 with RONs from different species. Stable NIH3T3 cells expressing human, monkey, or mouse RON were incubated in duplicate with different amounts of H5B14 followed by goat anti-human IgG coupled with FITC. Immunofluorescent intensities from individual samples were determined by flow cytometric analysis. Results are shown as the percentages of H5B14 specific binding to RON. The binding affinity (IC50) was calculated using the GraphPad Prism 7 software. [file 40425_2019_732_MOESM2_ESM.pdf]

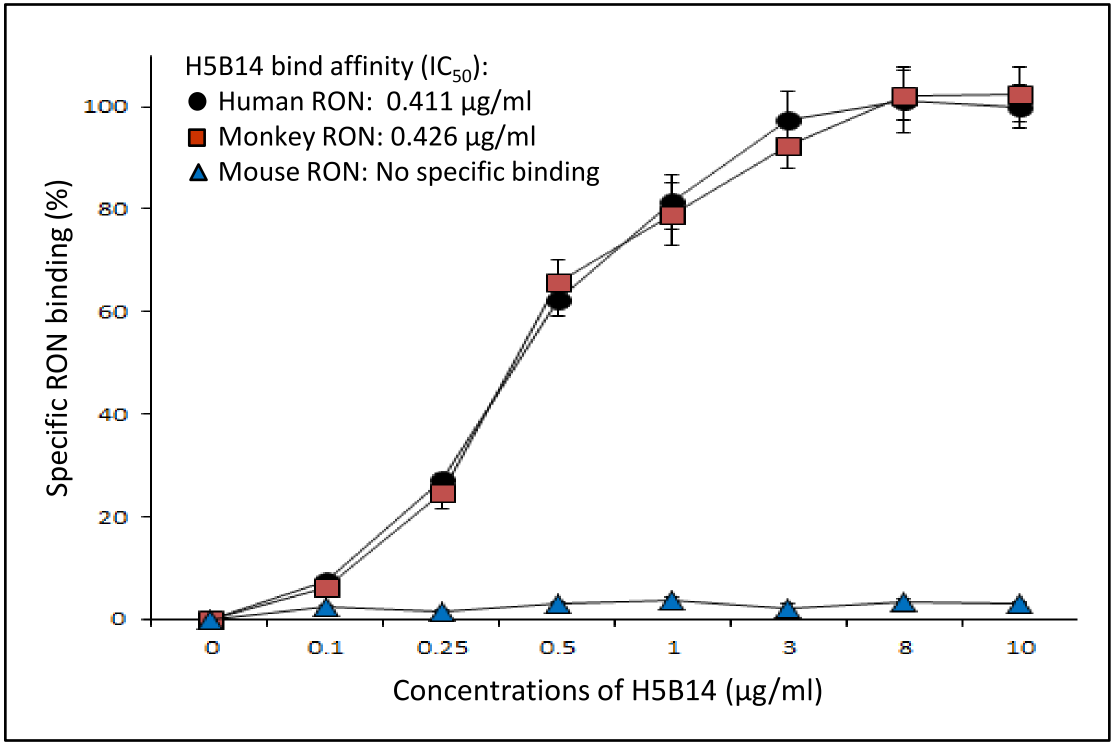

Figure S2 Interaction of H5B14 with RONs from different species. Stable NIH3T3 cells expressing human, monkey, or mouse RON were incubated in duplicate with different amounts of H5B14 followed by goat anti-human IgG coupled with FITC. Immunofluorescent intensities from individual samples were determined by flow cytometric analysis. Results are shown as the percentages of H5B14 specific binding to RON. The binding affinity (IC_{50}) was calculated using the GraphPad Prism 7 software.

Xiang-Min Tong, et al., Supplementary Figure 2
