# Supplementary material for: Therapeutic efficacy of a novel humanized antibody-drug conjugate recognizing plexin-semaphorin-integrin domain in the RON receptor for targeted cancer therapy
Source: J Immunother Cancer. 2019 Sep 13;7:250. doi: 10.1186/s40425-019-0732-8 (PMC6743155; doi:10.1186/s40425-019-0732-8)
Supplement: Supplementary file 3 — Additional file 3: Figure S3. Stability of H5B14-based ADCs in PBS. H5B14-MMAE and H5B14-DCM at 10 μg/ml were incubated with 1 ml PBS at room temperature for 28 days. Samples were collected at different time intervals and analyzed by HIC. Individual peaks with different numbers of MMAE or DCM conjugated to H5B14 were marked as P0 to P6. The average DAR combining P2, P4, and P6 for both ADCs were calculated accordingly [1–3]. [file 40425_2019_732_MOESM3_ESM.pdf]

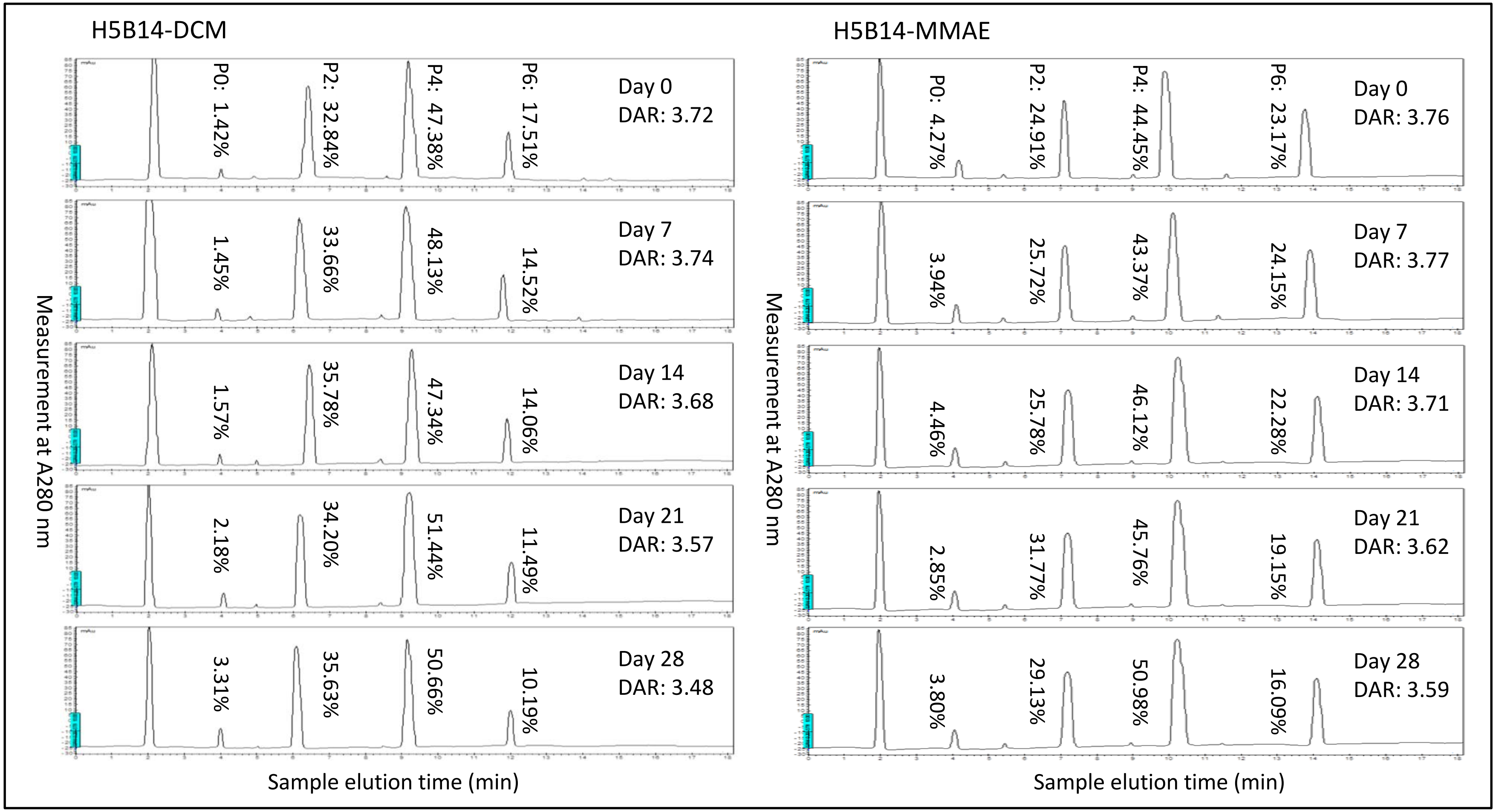

Figure S3 Stability of H5B14-based ADCs in PBS. H5B14-MMAE and H5B14-DCM at 10 µg/ml were incubated with 1 ml PBS at room temperature for 28 days. Samples were collected at different time intervals and analyzed by HIC. Individual peaks with different numbers of MMAE or DCM conjugated to H5B14 were marked as P0 to P6. The average DAR combining P2, P4, and P6 for both ADCs were calculated accordingly [1-3].

Xiang-Min Tong, et al., Supplementary Figure 3
