# Supplementary material for: Therapeutic efficacy of a novel humanized antibody-drug conjugate recognizing plexin-semaphorin-integrin domain in the RON receptor for targeted cancer therapy
Source: J Immunother Cancer. 2019 Sep 13;7:250. doi: 10.1186/s40425-019-0732-8 (PMC6743155; doi:10.1186/s40425-019-0732-8)
Supplement: Supplementary file 4 — Additional file 4: Figure S4. The concentration-dependent effect of H5B14-based ADCs on cell viability. A panel of fifteen cancer cell lines expressing variable levels of RON was used as the model. Cells at 8000 cells per well in a 96-well plate in triplicate were treated with different amounts of H5B14-MMAE (A) or H5B14-DCM (B) for 72 h. Cell viability was determined by the MTT assay. Zt/g4-MMAE or Zt/g4-DCM were used for comparison. [file 40425_2019_732_MOESM4_ESM.pdf]

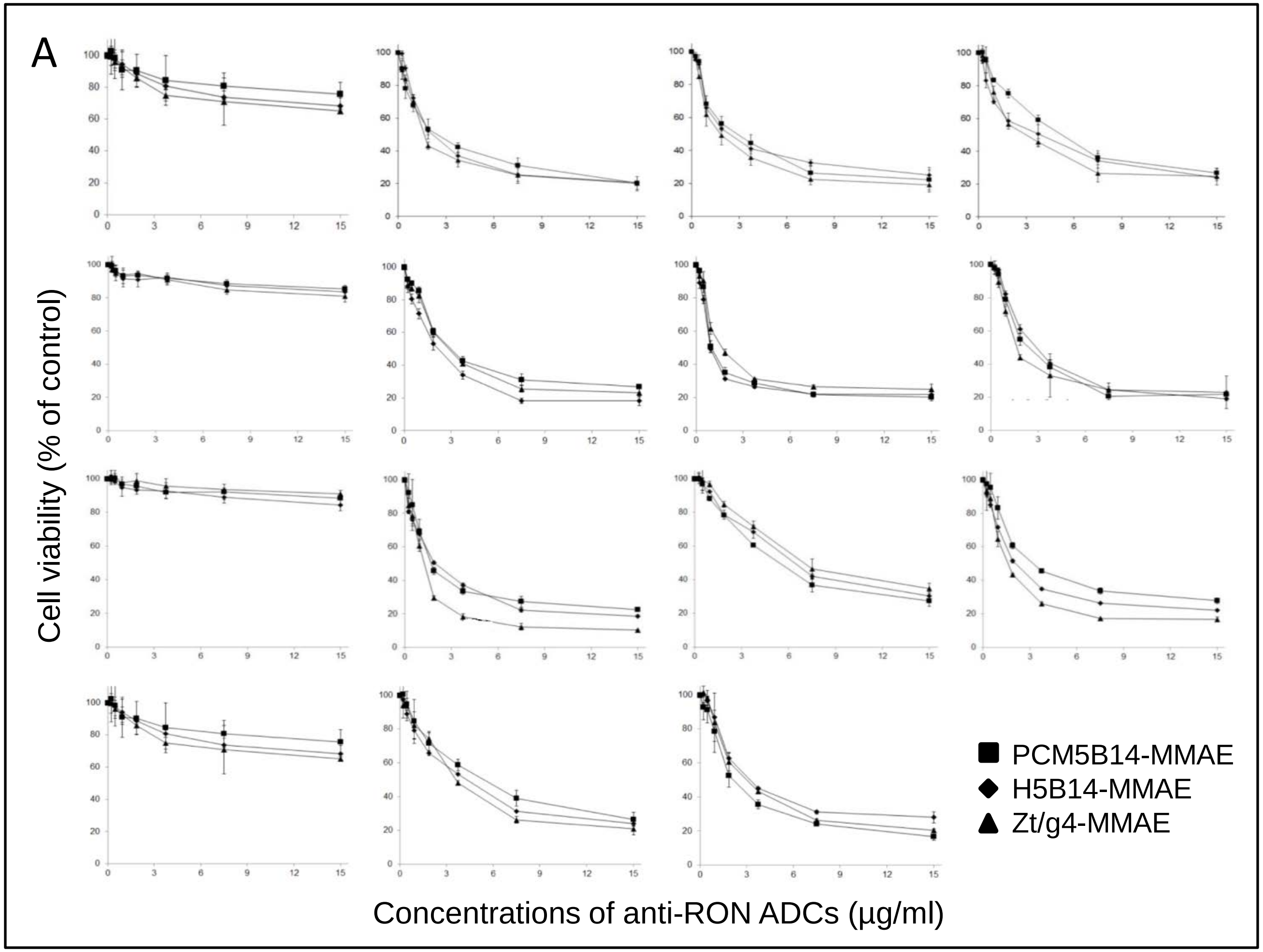

Xiang-Min Tong, et al., Supplementary Figure 4A

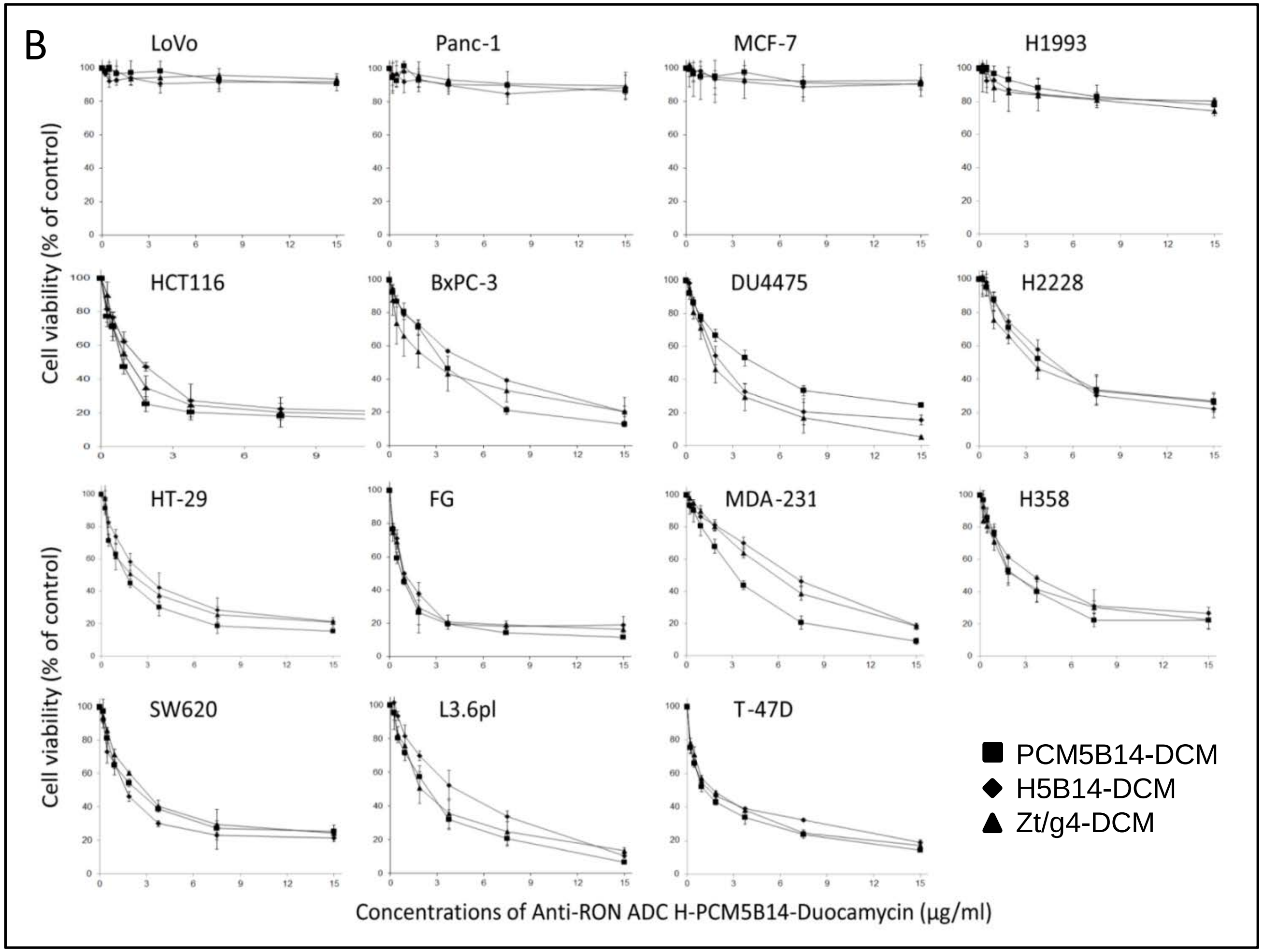

Figure S4 The concentration-dependent effect of H5B14-based ADCs on cell viability. A panel of fifteen cancer cell lines expressing variable levels of RON was used as the model. Cells at 8000 cells per well in a 96-well plate in triplicate were treated with different amounts of H5B14-MMAE (A) or H5B14-DCM (B) for 72h. Cell viability was determined by the MTT assay. Zt/g4-MMAE or Zt/g4-DCM were used for comparison.

Xiang-Min Tong, et al., Supplementary Figure 4B
