# Supplementary material for: Therapeutic efficacy of a novel humanized antibody-drug conjugate recognizing plexin-semaphorin-integrin domain in the RON receptor for targeted cancer therapy
Source: J Immunother Cancer. 2019 Sep 13;7:250. doi: 10.1186/s40425-019-0732-8 (PMC6743155; doi:10.1186/s40425-019-0732-8)
Supplement: Supplementary file 5 — Additional file 5: Figure S5. Effect of H5B14-based ADCs on mouse bodyweight. Female athymic nude mice (five mice per group) were injected with H5B14-MMAe or H5B14-DCM at 40, 60, 80, and 100 mg/kg in a single dose through the tail vein, respectively. Animals were monitored daily for activity, responsiveness, food consumption, and others. Individual mice were weighted every day to reach an average bodyweight for each group. All animals were sacrificed at the end of the study. [file 40425_2019_732_MOESM5_ESM.pdf]

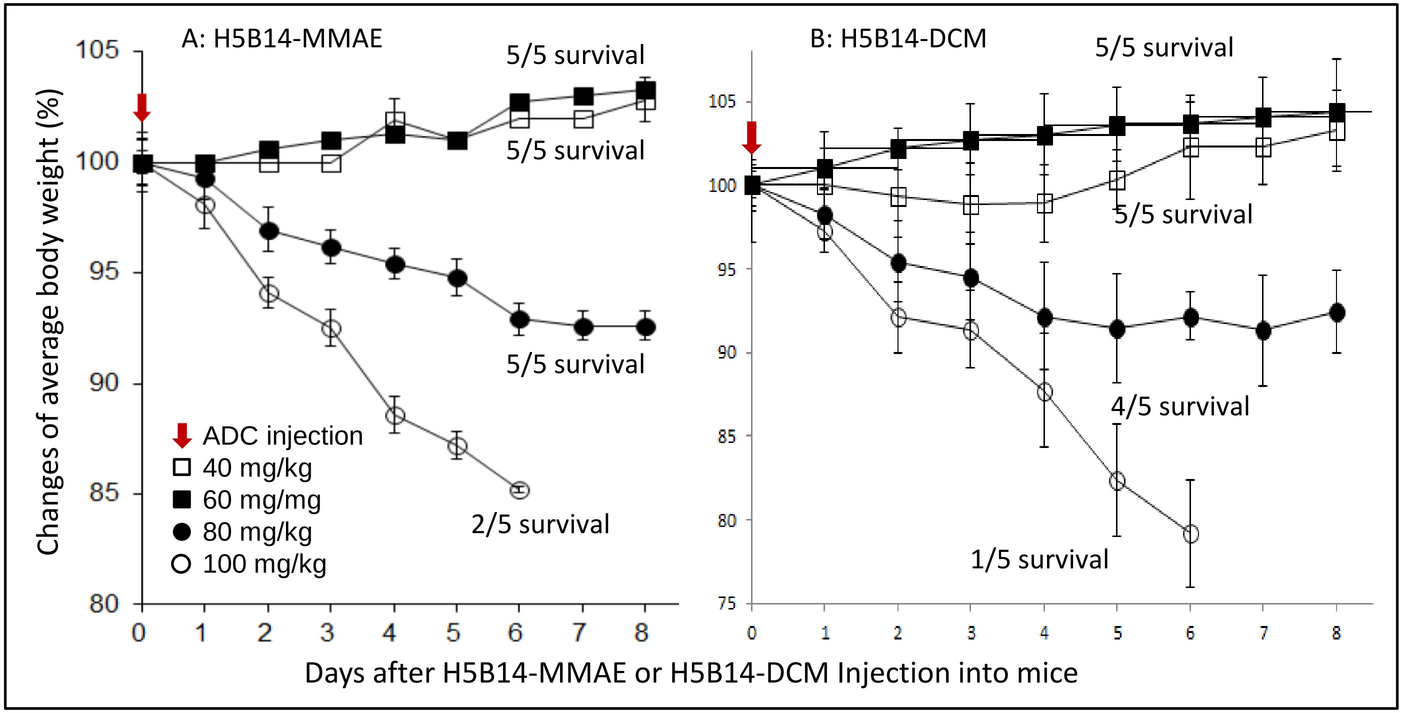

Figure S5 Effect of H5B14-based ADCs on mouse bodyweight. Female athymic nude mice (five mice per group) were injected with H5B14-MMAe or H5B14-DCM at 40, 60, 80, and 100 mg/kg in a single dose through the tail vein, respectively. Animals were monitored daily for activity, responsiveness, food consumption, and others. Individual mice were weighted every day to reach an average bodyweight for each group. All animals were sacrificed at the end of the study.

Xiang-Min Tong, et al., Supplementary Figure 5
