# Supplementary material for: Therapeutic efficacy of a novel humanized antibody-drug conjugate recognizing plexin-semaphorin-integrin domain in the RON receptor for targeted cancer therapy
Source: J Immunother Cancer. 2019 Sep 13;7:250. doi: 10.1186/s40425-019-0732-8 (PMC6743155; doi:10.1186/s40425-019-0732-8)
Supplement: Supplementary file 6 — Additional file 6: Table S1. Efficacy of H5B14-Mediated RON Internalization in Comparison with Other Anti-RON mAbs. [file 40425_2019_732_MOESM6_ESM.pdf]

Table S1 Efficacy of H5B14-Mediated RON Internalization in Comparison with Other Anti-RON mAbs

Cancer cell lines (RON receptors per cell)	IE_{50} values of different anti-RON mAbs in induction of cell surface RON internalization (hours)*					
	PCM5B14 (PSI domain	H5B14 (PSI domain)	Zt/g4 (Sema)	Zt/f12 (Sema)	Zt/c1 (IPT domain)	Zt/c11 (unknown)
**Colon LoVo (<100 ± 13)**	No effect	No effect	No effect	No effect	No effect	ND
**Colon HT-29 (18,793 ± 278)**	7.35 ± 0.33	5.32 ± 0.53	11.02 ±	19.11 ± 1.88	19.32 ± 0.94	No effect
**Colon DLD1 (4,480 ± 352)**	11.40 ± 1.42	10.48 ± 1.47	15.81 ± 2.44	21.75 ± 2.56	27.83 ± 1.11	ND
**Lung H1993 (2,152 ± 208)**	12.34 ± 1.26	13.51 ± 1.73	24.72 ± 3.84	20.61 ± 2.24	No effect	ND
**Lung H2228 (10,207 ± 278)**	9.43 ± 0.47	8.62 ± 0.68	9.84 ± 0.88	11.43 ± 1.67	16.78 ± 0.79	No effect
**Lung H358 (15,286 ± 366)**	7.04 ± 0.55	7.84 ± 0.69	7. 47 ± 0.63	13.47 ± 0.78	18.44 ± 1.37	No effect
**Pancreatic Panc-1 (<100 ± 4)**	No effect	No effect	No effect	No effect	No effect	ND
**Pancreatic L3.6pl (16,628 ± 245)**	6.68 ± 0.76	8.24 ± 0.61	14.14 ± 1.54	18.46 ± 1.22	18.82 ± 1.43	No effect
**Pancreatic BxPC-3 (10,214 ± 310)**	5.73 ± 0.65	7.52 ± 0.49	12.77 ± 0.82	14.56 ± 0.83	16.77 ± 2.18	Not effect
**Breast T-47D (15,756 ± 314)**	9.34 ± 0.84	8.6 ± 0.49	23.46 ± 2.88	17.58 ± 1.24	21. 34 ± 1.39	ND
**Average**	8.66 ± 2.36	8.77 ± 2.39	14.90 ± 6.23	17.12 ± 3.62	19.90 ± 3.83	No effect

*Individual cell lines (1 x 10^6 cells per culture dish in triplicate) were treated at 37°C with 5 µg/ml of different anti-RON mAbs. Cells were collected at different time points, washed with acidic buffer to remove cell surface-bound IgG and then incubated with anti-RON mAb Zt/f2 as previously described [1]. Cell surface immunofluorescence was analyzed by a flow cytometer using FITC-coupled goat anti-human or mouse IgG, respectively. Immunofluorescence from cells treated with anti-RON mAbs at 4°C was set as 100%. Internalization efficacy was calculated as previously described [1-3].
